# Complete Genome Sequence of Streptococcus mutans 27-3, an Active Extracellular Membrane Vesicle Producer

**DOI:** 10.1128/MRA.00166-21

**Published:** 2021-06-24

**Authors:** Xiaochang Huang, Justin Merritt, Zezhang Tom Wen

**Affiliations:** a Department of Oral and Craniofacial Biology, School of Dentistry, Louisiana State University Health, New Orleans, Louisiana, USA; b Department of Restorative Dentistry, Oregon Health and Science University, Portland, Oregon, USA; c Department of Molecular Microbiology and Immunology, Oregon Health and Science University, Portland, Oregon, USA; d Department of Microbiology, Immunology and Parasitology, School of Medicine, Louisiana State University Health, New Orleans, Louisiana, USA; University of Maryland School of Medicine

## Abstract

Here, we report the complete genome sequence of Streptococcus mutans 27-3. Isolated from a caries-active patient, 27-3 produces significantly more extracellular membrane vesicles than the commonly used laboratory strain UA159. This study provides useful information for comparative genomic analysis and better understanding of regulation of vesiculogenesis in this bacterium.

## ANNOUNCEMENT

Streptococcus mutans, a key etiological agent of human dental caries, actively releases membrane vesicles (EMVs) (1–4). 27-3 was isolated from a patient with active caries in Oklahoma City, OK, in 2006, and the glycerol stock was kept in a −80°C freezer (5). It grows similarly to UA159, but unlike UA159, 27-3 produces many more EMVs and possesses numerous particle-like structures around the cell envelope under transmission electron microscopy (5), although the nature and function of these structures await further investigation.

For genome sequencing, 27-3 was grown in brain heart infusion at 37°C in an aerobic chamber with 5% CO_2_ until the optical density at 600 nm (OD_600_) reached ≅0.4, and genomic DNA was extracted using the standard phenol-chloroform method (6, 7). For Illumina sequencing, genomic DNA was sheared by ultrasonication with fragment sizes of ∼300 to 500 bp, and the library was prepared and sequenced using a HiSeq 4000 platform. Raw reads were trimmed to remove adapters and low-quality sequences using Trimmomatic V0.36 with MINLEN:100 as the parameter (8). Consequently, 6,288,757 pairs of high-quality reads were left and used for further analysis. For PacBio sequencing, genomic DNA was sheared using a g-TUBE device with a fragment size of ∼6.0 kb, and SMRTbell DNA libraries were constructed and sequenced on a Sequel II system. The original sequencing results were processed using SMRTlink V9.0 with minLength 0 and minReadScore 0.8 as the parameters, and 248,086 PacBio subreads with an average of 6,356 bp were obtained. *De novo* assembly was performed using the genome assembler SPAdes V3.14.1 with default parameters (9). A single contig with average coverage as high as 824× was returned. To close the genome, the contig file was opened using Notepad++ (https://notepad-plus-plus.org/), identical sequences between the two ends of the contig were searched manually, and overlapping sequences were removed. To verify that the contig fully represents the total 27-3 genome, a relinearization contig was created by moving the first 10-kb sequence from the start position to the end, and it was mapped with the Illumina reads using Bowtie2 V2.4.1 (10). Overall, 99.37% of the short reads were aligned, indicative of complete assembly of the genome.

The genome of 27-3 is 1,978,522 bp long with a GC content of 37%. Annotation was carried out using the NCBI Prokaryotic Genome Annotation Pipeline (11), which predicted a total of 1,928 genes, including 1,792 coding sequences, 52 pseudogenes, and 84 RNAs. The average nucleotide identities (ANI) between the 27-3 genome and all available complete genomes in NCBI were compared using FastANI (12). 27-3 showed the most similarity to T8 (ANI, 99%), followed by UA140 (ANI, 98.9%). The genome was also analyzed for putative biosynthesis gene clusters (BGC) using antiSMASH V5.1.2 with default settings (13). Among the BGCs are those for mutacins and nonmutacin bacteriocins, type III polyketide synthase (T3PKS), and nonribosomal peptide synthesis (NRPS).

### Data availability.

The complete genome sequence of 27-3 has been deposited in GenBank. The BioSample number is SAMN16268312. The GenBank accession number for the genome is CP066294. The BioProject number is PRJNA665774. The Illumina read accession number is SRR14074425, and the PacBio read accession number is SRR13617002.
